# Clinical outcomes associated with anti-CD38-based retreatment in relapsed/refractory multiple myeloma: a systematic literature review

**DOI:** 10.3389/fonc.2025.1550644

**Published:** 2025-03-12

**Authors:** Francesca Gay, Elena Zamagni, Craig Emmitt Cole, Christof Scheid, Malin Hultcrantz, Justyna Chorazy, Ike Iheanacho, Anuja Pandey, Jacopo Bitetti, Natalie Boytsov, Molly Purser, Simon McNamara, Shinsuke Iida

**Affiliations:** ^1^ University Division of Hematology, Azienda Ospedaliero-Universitaria Città della Salute e della Scienza di Torino, Torino, Italy; ^2^ Department of Biotechnology and Health Science, University of Torino, Torino, Italy; ^3^ IRCCS Azienda Ospedaliero-Universitaria di Bologna, Istituto di Ematologia “Seràgnoli”, Bologna, Italy; ^4^ Dipartimento di Scienze Mediche e Chirurgiche, Università di Bologna, Bologna, Italy; ^5^ Department of Oncology, Karmanos Cancer Institute, Wayne State University, Detroit, MI, United States; ^6^ Division of Hematology and Oncology, Michigan State University, Lansing, MI, United States; ^7^ Department I of Medicine, University Hospital Cologne, Cologne, Germany; ^8^ Myeloma Service, Memorial Sloan Kettering Cancer Center, New York, NY, United States; ^9^ Evidera, London, United Kingdom; ^10^ Global Medical Affairs, GSK, Zug, Switzerland; ^11^ Value Evidence and Outcomes, GSK, Upper Providence, PA, United States; ^12^ Global Real World Evidence and Health Outcomes Research, GSK, Stevenage, United Kingdom; ^13^ Department of Hematology and Oncology, Nagoya City University Institute of Medical and Pharmaceutical Sciences, Nagoya, Japan

**Keywords:** anti-CD38, multiple myeloma, relapsed/refractory, systematic literature review, retreatment

## Abstract

**Introduction:**

Anti-CD38-based therapy has become a backbone regimen for the treatment of multiple myeloma (MM), approved in first-, second-, and third-line settings. The effectiveness of anti-CD38-based retreatment after an initial relapse on previous anti-CD38-based therapy is unclear. Here we present the results of a systematic literature review investigating the clinical outcomes of anti-CD38-based retreatment in patients with relapsed/refractory MM.

**Methods:**

Medline/Embase, congress publications, and other sources were searched (to December 8, 2023) for relevant articles in English and screened for eligibility criteria using the Population, Intervention, Comparator, Outcomes, Study Design (PICOS) framework, and data were then extracted for outcomes including progression-free survival (PFS), overall survival (OS), and overall response rate (ORR).

**Results:**

In total, 2938 records were identified from the initial Medline/Embase search and 11 were identified from other sources; 34 were eligible for inclusion, representing 24 studies (6 clinical [n=18–307] and 18 real-world evidence [RWE; n=19–583]). Where reported, median follow-up ranged from 1.9–43.0 months across 6 clinical and 8.7–53.0 months across 10 RWE studies. For clinical trials, anti-CD38-based retreatment resulted in a median PFS of 1.0–2.8 months in all but one trial (19.4 months), a median OS of 10.7–19.1 months (not reached in one trial), and ORRs of 0–75%. RWE studies reported a median PFS of 1.5–8.4 months, a median OS of 8.4–19.0 months (not reached in one study), and ORRs of 24.6–90.0%.

**Discussion:**

Findings from this systematic literature review indicate that clinical outcomes with anti-CD38-based retreatment are variable and offer limited clinical benefit in patients with relapsed/refractory MM, including in those refractory to anti-CD38-based treatment.

## 1 Introduction

Multiple myeloma (MM) is a malignant plasma cell disorder that accounts for 1.8% of all new cancer cases and 2.0% of all cancer-related deaths in the US, with an estimated 5-year survival rate of 61.1% (1). Newly diagnosed MM cases are assessed for transplant eligibility, which considers age, fitness, and comorbidities and helps to assign suitable first-line therapies based on current MM treatment guidelines (2, 3). Novel combinations, particularly quadruplet regimens, have emerged as frontline options in patients who were transplant ineligible and, more recently, transplant eligible, resulting in improved progression-free survival (PFS) and overall survival (OS) (4–6). These regimens are generally composed of a monoclonal antibody targeting CD38 (daratumumab or isatuximab) in combination with a proteasome inhibitor and/or immunomodulatory drug, and a steroid.

With the use of frontline combination regimens, patients become exposed and/or refractory to multiple effective drug classes early on, limiting treatment options in the relapsed/refractory multiple myeloma (RRMM) setting. In particular, the proportion of patients who are anti-CD38-refractory at first relapse is likely to increase, given the clinical efficacy shown with first-line anti-CD38 combination regimens in phase III trials (5–9). Currently, the anti-CD38 agent daratumumab is approved in combination regimens as first-line treatment for both patients who are transplant eligible and ineligible and in combinations or as monotherapy for patients with RRMM, while isatuximab is approved in combination regimens as first-line treatment for patients who are transplant ineligible and as second- or later-line treatment for patients with RRMM (10, 11).

With subsequent therapy options that have distinct mechanisms of action becoming more limited due to previous exposure and/or refractoriness, retreatment with anti-CD38 agents may be more frequently considered (12). Indeed, anti-CD38 retreatment has become relatively common in patients exposed to multiple drug classes, as shown in a real-world study, where 36% of patients exposed to two prior therapies and 48% of patients with triple-refractory MM were retreated with daratumumab (13). At present, the evidence supporting anti-CD38-based retreatment in patients who are anti-CD38 exposed or refractory is unclear and treatment guidelines generally do not recommend retreatment when a patient is considered refractory to the same agent (14, 15).

To understand the impact of anti-CD38 retreatment on patient outcomes, we conducted a systematic literature review (SLR) that aimed to identify, summarize, and draw insights from data on the clinical outcomes of anti-CD38-based retreatment in patients with RRMM.

## 2 Methods

### 2.1 Study design and search strategy

This SLR was conducted in adherence with Preferred Reporting Items for Systematic reviews and Meta-Analyses (PRISMA) guidelines (16, 17). Medline, Medline In-Process, and Embase electronic databases were searched for articles published in English from database inception to December 8, 2023. The proceedings from the following five pre-selected annual conferences were also searched (from 2016–2023): The American Association for Cancer Research, the American Society of Clinical Oncology, the American Society of Hematology, the European Hematology Association, and the European Society for Medical Oncology. The methodology for the Medline and Embase search is detailed in **Supplementary Table S1**. To supplement these searches, ClinicalTrials.gov was reviewed for ongoing clinical trials and trials with data not reported elsewhere, as were the bibliographies of relevant, recently published SLRs for any additional articles of relevance.

### 2.2 Selection criteria

The Population, Intervention, Comparator, Outcomes, Study Design (PICOS) framework was used to apply the SLR eligibility criteria (18), and all PICOS inclusion criteria were required to be met for studies to be included in the analysis (**Supplementary Table S2**). Studies had to include adults with RRMM who were previously treated and retreated with an anti-CD38-based therapy (daratumumab or isatuximab). For studies with mixed patient populations, ≥80% of patients must have been anti-CD38-retreated or data for the patients who were retreated with anti-CD38s had to be reported separately as a subgroup; studies must have reported results for ≥10 patients who were anti-CD38-retreated overall. Studies must have reported at least one of the outcomes of interest with anti-CD38-based retreatment including PFS, OS, time to progression, overall/objective response rate (ORR), complete response (CR) rate, very good partial response (VGPR) rate, and partial response (PR) rate. The main outcome of interest was PFS. Eligible study designs included randomized controlled trials (RCTs), single-arm trials, non-randomized trials, and observational/real-world evidence (RWE) studies. Case reports, qualitative studies, pharmacodynamic/pharmacokinetic studies, genetic studies, cellular or molecular studies, network meta-analyses, and economic evaluations were excluded. Whilst reference lists of relevant SLRs were used for trial identification, they were excluded as discrete studies. Full-text peer-reviewed original research articles, clinical trial records, and conference abstracts were included. Narrative reviews, editorials, protocols, guidelines, letters not reporting original research, errata, notes, or comments were excluded.

### 2.3 Study selection, data extraction, and quality assessment

All publication titles and abstracts were initially screened for eligibility using the Nested Knowledge platform, an internet-based program that incorporates artificial intelligence screening capabilities and facilitates collaboration among reviewers during the study selection process (19). The first screening was performed by a human reviewer, while the second screening was performed by artificial intelligence for 85% of abstracts and by a human reviewer for 15% of abstracts. Any conflicts were resolved by a third independent human reviewer.

Full-text screening was carried out by two independent human reviewers. Data were extracted by a single investigator, each data point was then validated by a second senior investigator, and any conflicts were resolved through discussion with a third investigator. Key data extracted included study characteristics (e.g., design, location, size, population, objectives, inclusion/exclusion criteria), patient characteristics (e.g., age, sex, disease stage, prior lines of therapy, high-risk cytogenetics, and refractoriness), treatment characteristics (e.g., dosing regimen, route of administration, duration of treatment, concomitant medications), and analysis outcomes. The extracted evidence was assessed using narrative synthesis. A quality assessment of studies included in the SLR was also performed using the Mixed Methods Appraisal Tool (MMAT) (20).

### 2.4 Ethics approval statement

Due to the nature of this analysis, neither ethics committee nor institutional review board approval was needed as no patient participation or consent was required and no personally identifiable information was used, stored, or disclosed.

## 3 Results

### 3.1 Literature search

Of the 2938 records identified from the initial Medline/Embase searches, 130 were duplicates and excluded, 2621 were excluded during title/abstract eligibility screening, and 164 were excluded during full-text screening. To the 23 remaining records, 11 were added from other sources (nine from conference proceedings, one from ClinicalTrials.gov, and one from SLR bibliography searches). The final 34 records collectively represented data from 24 studies, which were included in the SLR (**Supplementary Figure S1**).

### 3.2 Quality assessment

In total, six clinical trials (two RCTs and four single-arm trials) and 18 RWE studies were included in the analysis; 16 studies had sufficient information to perform the MMAT assessment. All of these studies were of sufficient quality to address our research questions, and included appropriate sampling strategies, patient populations, outcomes, and statistical analyses. Randomization was appropriately performed in the RCTs, both of which had complete outcomes data. There were no substantive concerns about the quality of the studies or data.

### 3.3 Study characteristics

Characteristics of the studies are summarized in **Tables 1**, **2**. Of the RWE studies, one was a prospective study (EMMY (21)) and 17 were retrospective observational studies. Across clinical trials and RWE studies, there was considerable variation in their characteristics, including number of patients in the overall study population (ranging from 18–307 in clinical trials and 19–583 in RWE studies), treatments used, and the median follow-up periods (ranging from 1.9–53 months). Similarly, there was substantial variation in key patient characteristics, with median prior lines of therapy ranging from 3–7, 11–81% of patients having high-risk cytogenetics, and large variation in refractoriness (3–100%, double-refractory; 11–92%, triple-refractory; 7–67%, penta-refractory; 28–100%, daratumumab-refractory, where reported). Additional key patient characteristics are presented in **Supplementary Table S3**.

**Table 1:** Summary of included clinical trial publications.

Clinical trial type	Publication	Anti-CD38-based retreatment regimen*	Median follow-up duration (range), months	Median (range) prior lines of therapy	% refractory (overall population)
RCT					
NCT03194867 Phase I/II	Lesokhin, 2023 (25)	Isa ± Cemiplimab	10.0 (8.5–10.9)^‡^	>3	100% Dara ref
ICARIA-MM NCT02990338 Phase III	Richardson, 2022 (27, 57)	Dara regimens^†^	35.3 (33.5–37.4)^§^	3 (2–4)^§^	NR
	Perrot, 2021 (58)				
	Richardson, 2021 (59)				
Single-arm clinical trials					
TRIMM-2 NCT04108195 Phase I	Bahlis, 2023 (22)	Dara + Talq	11.5 (1.0–27.3)	>3	77% anti-CD38 ref
NCT02751255 Phase I/II	Frerichs, 2021 (23)	Dara + ATRA	43	5 (3–12)	100% Dara ref
FUSION-MM-005 NCT03000452 Phase II	Frerichs, 2021 (24)	Dara + Durva	2.9 (0.13–5.8)	5 (5–16)	100% Dara ref
	Clinicaltrials.gov (60)				
NCT02514668 Phase I/II	Mikhael, 2021 (26)	Isa	1.9 (0.8–17.0) (4.7 [0.4–18.5] for OS)	7 (2–14)	100% Dara ref

*Only anti-CD38-based regimens used for retreatment are shown; therefore, subgroup details are presented for some studies. ^†^Detailed here is the subgroup analysis of patients treated with Dara after receiving Isa + Pom + Dex or Pom + Dex. ^‡^Median (IQR). ^§^Median (95% CI).

ATRA, all-trans retinoic acid; CI, confidence interval; Dara, daratumumab; Dex, dexamethasone; Durva, durvalumab; IQR, interquartile range; Isa, isatuximab; NR, not reported; OS, overall survival; Pom, pomalidomide; RCT, randomized controlled trial; ref, refractory; Talq, talquetamab.

**Table 2:** Summary of included RWE publications.

RWE study type	Publication	Anti-CD38-based retreatment regimen*	Median follow-up duration (range), months	Median (range) prior lines of therapy	% refractory (overall population)
Retrospective	Abdallah, 2023 (37)	Dara-based therapy	19.5 (10.3–25.9)	NR	100% Dara ref
Retrospective	Kostopoulos, 2023 (32)	Dara + IMD	NR	3 (1–16)	100% Dara ref
	Fotiou, 2021 (61)				
	Fotiou, 2020 (62)			4 (1–16)	NR
Retrospective	Kastritis, 2023 (63)	Anti-CD38-based retreatment NS	NR	3 (1–11)	100% anti-CD38 ref
	Kastritis, 2022 (30)			2 (1–10)	
Retrospective	Kikuchi, 2023 (31)	Isa	8.7 (0.1–25.0)	4 (1–8)	72% Dara ref
Retrospective	Perez de Acha, 2023 (40)	Dara- and Isa-based therapies	≤53 (NR)	5 (2–11)	90% Dara ref 42% penta ref
IMAGE study Retrospective	Decaux, 2022 (29)	Isa + Pom + Dex: Dara ref vs Dara non-ref	14.2 (NR)	NR	28% Dara ref
Retrospective	Girvan, 2022 (64)	Dara-based therapy	NR	NR	100% Dara ref
EMMY study Prospective	Hulin, 2022 (21)	Anti-CD38-based retreatment NS	NR	NR	73% anti-CD38 ref
Retrospective	Leblanc, 2022 (39)	Anti-CD38-based retreatment NS	21 (NR)	4 (2–10)	NR
Retrospective	Reyes, 2022 (42)	Anti-CD38-based retreatment NS	21.3 (NR)	7 (1–14)	NR
Retrospective	Zhou, 2022 (35)	Dara, Carf-based regimen	NR	5 (2–12)	97% Dara ref 55% penta-ref
Retrospective	Szabo, 2022 (43)	Dara	9.2 (1.8–17.6)^†^	3 (0–15)	NR
	Szabo, 2021 (65)				
Retrospective	Atrash, 2021 (38)	Dara	NR	NR	NR
Retrospective	Regidor, 2021 (41)	Ven + Bor + Dara + Dex	NR	7 (2–16)	NR
Retrospective	Yashar, 2021 (34)	Anti-CD38-based retreatment NS	NR	NR	NR
MAMMOTH study Retrospective	Costa, 2021 (28)	Dara-based regimen	NR	5 (3–17)	100% triple ref
	Gandhi, 2019 (36)		10.6 (1.9–42.3)	5 (2–17)	100% anti-CD38 ref
Retrospective	Zhou, 2020 (44)	Pom + Bor + Dox + Dex + Dara	NR	4 (1–10)	100% penta-ref
Retrospective	Nooka, 2019 (33)	Dara + Pom + Dex	41 (NR)	6.5 (3–13)	100% Dara ref
	Nooka, 2016 (45)	Dara + Pom + Dex	Dara and Pom naïve: 14 months Dara and Pom ref: 5 months Dara ref: 3 months	Dara and Pom naive: 3 (1–7) Dara and Pom ref: 6.5 (3–13) Dara ref: 6 (3–13)	29% Dara and Pom ref

*Only anti-CD38-based regimens used for retreatment are shown; therefore, subgroup details are presented for some studies. †Median (IQR).

Bor, bortezomib; Carf, carfilzomib; Dara, daratumumab; Dex, dexamethasone; Dox, doxorubicin; IMD, immunomodulatory drug; IQR, interquartile range; Isa, isatuximab; NR, not reported/not reached; Pom, pomalidomide; ref, refractory.

### 3.4 Retreatment outcomes from clinical trials

**Figure 1:**
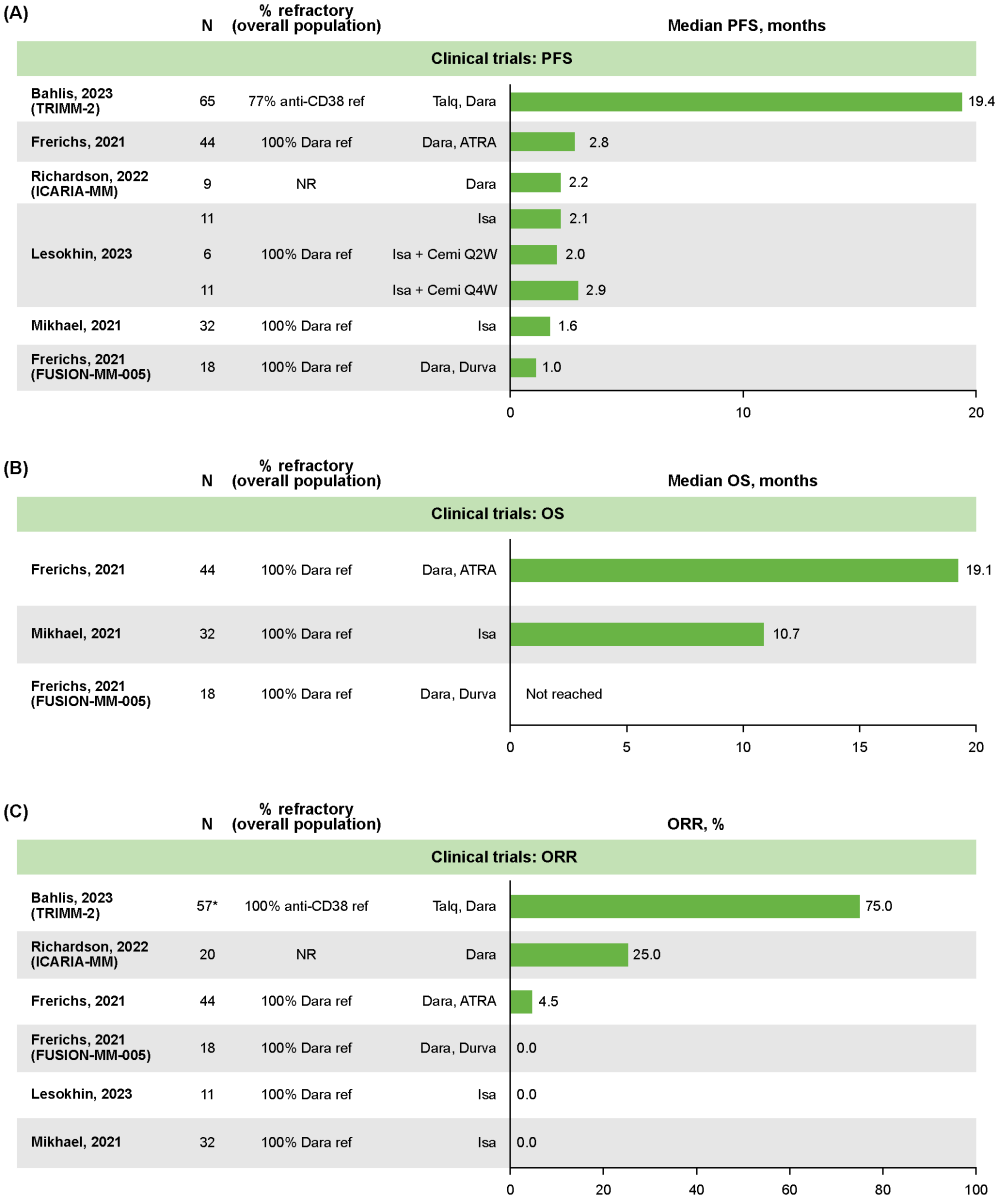
Clinical trial outcomes in patients with RRMM following retreatment with anti CD38-based therapy: **(A)** median PFS, **(B)** median OS, **(C)** ORR. *The N for TRIMM-2 was extrapolated from 88% of 65 patients who were previously exposed to an anti-CD38 drug. ATRA, all-trans retinoic acid; Cemi, cemiplimab; Dara, daratumumab; Durva, durvalumab; Isa, isatuximab; NR, not reported; ORR, overall response rate; OS, overall survival; PFS, progression-free survival; QxW, every x weeks; ref, refractory; RRMM, relapsed/refractory multiple myeloma; Talq, talquetamab.

Median PFS data were reported for all six clinical trials (22–27), with treatment groups ranging from 6–65 patients (**Figure 1A**). Overall, the median PFS was <3 months in all studies except the TRIMM-2 trial. TRIMM-2 evaluated the novel therapy talquetamab in combination with daratumumab, and reported a median PFS of 19.4 months (95% confidence interval [CI] not reported [NR]) in 65 patients after a median follow-up of 11.5 months (22). The ICARIA-MM study (median follow-up 35.3 months for overall study population) reported a median PFS of 2.2 months (95% CI: 0.1–7.6) for nine patients with RRMM who received an isatuximab regimen followed by daratumumab as the first line of subsequent therapy (27). In contrast, the median PFS was 4.2 months (95% CI: 2.8–4.8) for 82 patients who received an isatuximab regimen followed by a non-daratumumab therapy (27).

OS was reported in three clinical trials, with median not reached in one study of daratumumab plus durvalumab (N=18) (24), and the remaining two reporting medians of 10.7 months (95% CI: 8.0–19.0; N=32; median follow-up 4.7 months; isatuximab monotherapy [N=32]) (26) and 19.1 months (95% CI: 15.0–23.1; N=44; median follow-up 43 months; daratumumab plus all-trans retinoic acid [N=44]; **Figure 1B**) (24).

ORR data were reported for all six clinical trials (22–27), with treatment groups ranging from 11–57 patients. The ORR was 0% in three trials, 4.5% (n=NR/44) in a study of daratumumab plus all-trans retinoic acid in patients who were daratumumab refractory (NCT02751255), 25% (n=5/20) in ICARIA-MM, and 75% (n=NR/NR) in TRIMM-2 (**Figure 1C**) (22, 23, 27).

### 3.5 Retreatment outcomes from RWE studies

**Figure 2:**
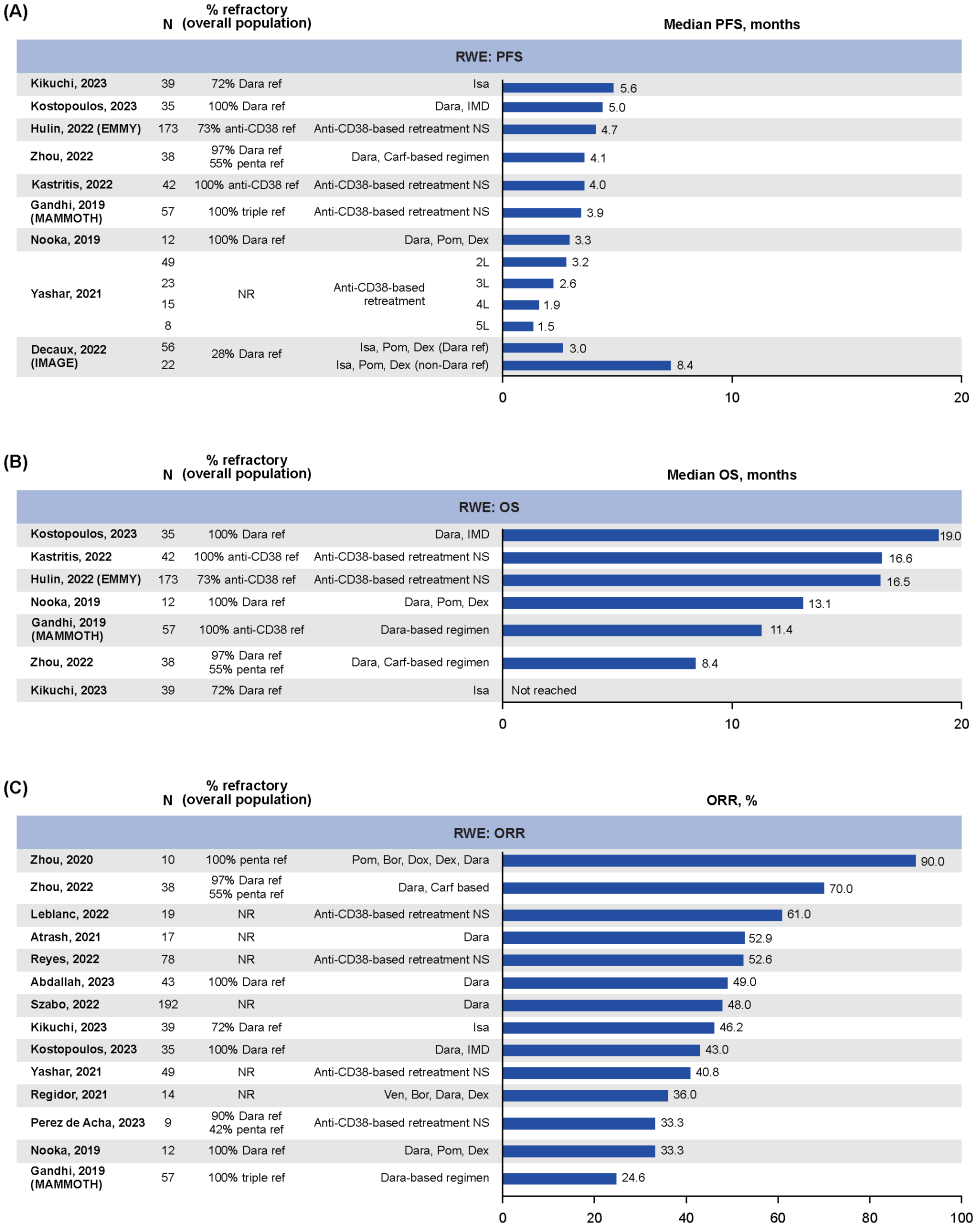
RWE for outcomes in patients with RRMM following retreatment with anti-CD38-based therapy: **(A)** median PFS, **(B)** median OS, **(C)** ORR. 2/3/4/5L, second/third/fourth/fifth line of therapy; Carf, carfilzomib; Dara, daratumumab; Dex, dexamethasone; Dox, doxorubicin; IMD, immunomodulatory drug; Isa, isatuximab; NR, not reported; ORR, overall response rate; OS, overall survival; PFS, progression-free survival; Pom, pomalidomide; ref, refractory; RRMM, relapsed/refractory multiple myeloma; Ven, venetoclax; RWE, real-world evidence.

Median PFS data were reported in nine RWE studies (21, 28–35), ranging from 1.5 months (95% CI: NR; N=8; median follow-up NR) for patients with five lines of anti-CD38 exposure to 8.4 months (95% CI: 2.8–not estimable; N=22; median follow-up 14.2 months) in patients exposed but not refractory to daratumumab who received isatuximab plus pomalidomide and dexamethasone in the IMAGE study (**Figure 2A**) (29, 34). Overall, median PFS tended to be shorter in studies with >95% of patients who were anti-CD38-refractory, ranging from 3.3 months (95% CI: 0.0–6.9; N=12; median follow-up NR) to 5.0 months (95% CI: 1.5–8.4; N=35; median follow-up NR) (32, 33, 35, 36). In the IMAGE study of patients with RRMM who received isatuximab plus pomalidomide and dexamethasone, those who were refractory to daratumumab (median follow-up 14.2 months) had a median PFS of 3.0 months (95% CI: 2.4–4.8; N=56), while those who were daratumumab naïve had a median PFS of 16.6 months (95% CI: 13.2–not reached; N=215; **Supplementary Figure S2**) (29).

Median OS data for anti-CD38-based retreatment was reported in seven RWE studies (21, 30–33, 35, 36), with treatment groups ranging from 12–173 patients (**Figure 2B**). Overall, the median OS ranged from 8.4 months (95% CI: 6.7–10.0; N=38; median follow-up NR) in patients who were heavily pretreated (55% penta-refractory) who had received daratumumab, carfilzomib, dexamethasone, thalidomide, cisplatin, doxorubicin, cyclophosphamide, and etoposide (Dara-KDT-P[A]CE) (35) to 19.0 months (95% CI: 13.5–24.5; N=35; median follow-up NR) in patients treated with daratumumab plus an immunomodulatory drug who were refractory to both (32).

ORR data were reported in 14 RWE studies (31–44), with treatment groups ranging from 9 to 192 patients (**Figure 2C**). The ORRs ranged from 24.6% (35) to 90.0% (28) (**Figure 2C**) (33, 36, 40, 41). Best overall response was reported in 11 RWE studies (31–38, 40, 43, 44), with treatment groups ranging from 13–192 patients per treatment group, and showed that ORR was predominantly driven by PR/VGPR, with rates of CR or better reported as 0% in six studies and ranging from 2% (n=1/49) (34) to 20.5% (n=8/39) (31) in the remaining five (**Supplementary Figure S3A**). Of note, the study that reported an ORR of 90% was in a small subgroup of patients who were penta-refractory (n=10) who were treated with pomalidomide, bortezomib, doxorubicin, dexamethasone, and daratumumab (44).

ORR data for patients receiving anti-CD38-based retreatment compared with patients who were anti-CD38 naïve were reported in three RWE studies, with treatment groups ranging from 19–49 patients (**Supplementary Figure S3B**) (34, 37, 45). For two of these studies, ORRs were lower with retreatment; 49% vs. 65% for daratumumab-based treatment in patients who were daratumumab-refractory vs. naïve (37), and 89.0% vs. 38.5% vs. 33.0% for daratumumab plus pomalidomide and dexamethasone in patients who were daratumumab/pomalidomide naïve vs. daratumumab refractory vs. daratumumab/pomalidomide refractory (45) (**Supplementary Figure S3B**) (45).

Only one study reported time between prior daratumumab-based therapy to retreatment (37). In this RWE study, the median time to retreatment was 1.25 months (range 0.25–25; n=21) for patients who responded to retreatment and 0.25 months (range 0.25–39; n=22) for patients who did not respond to retreatment (37).

## 4 Discussion

In recent years, novel combination therapies have improved the outcomes for patients with MM. In particular, the use of anti-CD38 combination therapies as first-line treatment for MM has improved responses and survival compared with historical controls (5–8, 46). However, as patients inevitably relapse or become refractory to early lines of therapy, it is important to evaluate the effectiveness of retreatment with approved RRMM therapies (especially anti-CD38-based regimens) to optimize treatment selection and sequencing. To our knowledge, this is the first SLR to examine retreatment with anti-CD38-based regimens.

Overall, these data suggest limited clinical benefit with anti-CD38-based retreatment (especially in later lines of therapy). Median PFS was <3 months across all clinical trials except for TRIMM-2, and <9 months across all RWE studies. For studies with adequate follow-up to report median OS, medians were <20 months across all trials (both clinical and RWE). ORR was <5% in all but two clinical trials (TRIMM-2 and ICARIA-MM) (14, 22). The TRIMM-2 study, which included 65 patients (88% anti-CD38 exposed and 77% refractory), showed a substantially longer PFS (19.4 months) and greater ORR (75%) than other studies (22). However, the benefits of this unlicensed combination are difficult to attribute specifically to anti-CD38-based retreatment rather than simply to the bispecific antibody talquetamab, a novel CD3- and GPRC5D-targeting agent. Indeed, as a monotherapy, talquetamab demonstrated an ORR of 73–74% in the pivotal cohorts of a phase II study of patients with RRMM who were heavily pretreated (23–29% were penta-refractory in the relevant cohorts) (22, 47, 48). Further randomized studies of anti-CD38 combination therapies (including with talquetamab) vs. anti-CD38 monoclonal antibody free regimens in patients with RRMM are needed to clarify the independent efficacy of each drug component.

There was also high variability in ORR for RWE studies (range 25–90% across 14 studies). Variability in outcome measures is likely due to heterogeneity in patient populations (e.g., the presence or absence of high-risk MM features such as extramedullary disease), small sample sizes, and that most studies were conducted in later lines of therapy (>3) where the composition of combination regimens can vary widely (49). It is interesting to note that for the 11 RWE studies that reported best overall response, the ORR appeared to be primarily driven not just by PR but also VGPR. However, rates of CR were low, and further evidence on durability of responses is needed.

The phase II LYNX study investigated retreatment with daratumumab after up to three lines of therapy, but did not meet the cutoff date for inclusion in this SLR (49). In this study, patients who received 1–3 prior therapy lines, one of which contained daratumumab, were randomized to receive daratumumab plus carfilzomib and dexamethasone or carfilzomib plus dexamethasone, and the primary endpoint was rate of VGPR or better. VGPR or better was achieved in 48.8% (95% CI: 35.1–62.6) of patients in the daratumumab plus carfilzomib and dexamethasone arm vs. 46.2% (95% CI: 32.3–60.4) in the carfilzomib plus dexamethasone arm; the secondary endpoint of PFS was also not notably different between the arms (median 10.7 vs. 10.6 months), and the study was terminated early due to futility (49). The results of LYNX are consistent with the data in this SLR, suggesting that anti-CD38 retreatment confers no clear benefit in patients with RRMM.

One strength of this SLR is that it employed a robust protocol and search strategy, and the screening process ensured that only studies that met PICOS criteria were included. However, as with all studies, there were some limitations that should be considered when interpreting the results. Data availability was limited in some studies; outcomes were reported in few studies and many had small patient populations. There was also considerable variation in the follow-up periods between studies, which likely impacted the outcomes reported. Moreover, the majority of anti-CD38-based retreatment studies were retrospective RWE studies, single-arm trials, or subgroup analyses, which further limited the strength of the analysis. Findings from retrospective studies can be limited by selection bias and confounding factors. Therefore, future prospective studies are needed to validate the results reported in the RWE studies. In addition, the variety of retreatment regimens used across the studies (as well as the heterogeneity in study design, patient populations and characteristics, and outcomes assessed, especially for RWE studies) made it even more challenging to interpret outcomes and response data from the perspective of anti-CD38 retreatment effects, as other agents may have contributed to clinical activity. Considering the variability across patient populations, additional reporting of outcomes by subgroups in future studies would be useful to explore differences in the efficacy of anti-CD38 retreatment based on baseline clinical characteristics. Only one RWE study reported the time to anti-CD38 retreatment, limiting the interpretation of impact of time to treatment (37). Future clinical investigations should prospectively consider the efficacy of retreatment started within six months of anti-CD38 refractoriness vs more than six months, given that the expression level of CD38 is reported to be restored after 6 months, which may offer improved clinical response (40, 50).

Several ongoing trials are evaluating anti-CD38 therapy in combination regimens, including with agents that target B-cell maturation antigen; the bispecific antibodies teclistamab and talquetamab are being assessed with daratumumab for RRMM, while ciltacabtagene autoleucel, a chimeric antigen receptor T-cell treatment, is being assessed with daratumumab for newly-diagnosed MM (51–53). As mentioned in this SLR, the talquetamab plus daratumumab combination from the TRIMM-2 trial has already produced promising results (22), despite the difficulties in clarifying the role of a drug with a new mechanism of action vs the impact of retreatment with anti-CD38 monoclonal antibodies. Combination studies, including studies with anti-CD38 sparing regimens and immunotherapies (54–56), may further inform strategies to overcome variable outcomes in anti-CD38 refractory patients.

In summary, the findings from this SLR indicate that retreatment with current anti-CD38-based regimens offers only a limited clinical benefit in patients with RRMM, with shorter PFS shown in studies with higher rates of patients who are anti-CD38 refractory, and as such anti-CD38-based retreatment remains an investigational option only. The advent of novel and complementary anti-CD38-based combination therapies as well as non-anti-CD38 regimens may help to address this issue, but further clinical studies specifically designed to assess the efficacy and effectiveness of any novel retreatment combinations would be required.

## Data Availability

The original contributions presented in the study are included in the article/**Supplementary Material**. Further inquiries can be directed to the corresponding author.
