# Supplementary material for: Clinical outcomes associated with anti-CD38-based retreatment in relapsed/refractory multiple myeloma: a systematic literature review
Source: Front Oncol. 2025 Mar 12;15:1550644. doi: 10.3389/fonc.2025.1550644 (PMC11938063; doi:10.3389/fonc.2025.1550644)
Supplement: Supplementary file 1 [file DataSheet1.pdf]

Supplementary material

Supplementary Table 1. Medline and Embase search algorithm.

Search number	Search terms	Results
1	(exp myeloma/ and multiple.mp.) or myelom*.mp. or exp plasmacytoma/ or (plasm*.mp. and exp cell/ and myelom*.mp.) or (exp plasma/ and exp cell/ and exp leukemia/) or (plasma* adj3 neoplas*).ti,ab.	259,899
2	daratumumab/	6809
3	(daratumumab or dalinvi or darasarex or darzalex or hlx-15 or hlx15 or humax-CD38 or jnj-54767414 or jnj54767414 or anticd38 or anti-cd38 or anti-cd-38).ti,ab.	7668
4	isatuximab/	1057
5	(isatuximab or sarclisa or sar 650984 or sar650984).ti,ab.	812
6	"ADP ribosyl cyclase/cyclic ADP ribose hydrolase 1"/ or ADP-ribosyl Cyclase 1/	11,865
7	(cd38$ or cyclic ADP ribose hydrolase or ADP ribosyl cyclase).ti,ab.	29,089
8	or/2-7	38,018
9	1 and 8	11,341
10	exp randomized controlled trial/ or exp RANDOMIZATION/ or random*.ti,ab. or 'rct'.ti,ab. or 'controlled trial'.ti,ab. or 'clinical trial'.ti,ab. or 'trial'.ti,ab. or exp Single Blind Procedure/ or exp Double Blind Procedure/ or exp Crossover Procedure/ or 'cross over'.ti,ab. or 'crossover'.ti,ab. or exp PLACEBO/ or 'placebo'.ti,ab. or (doubl* and blind*).ti,ab. or (singl* and blind*).ti,ab. or ('open' and label*).ti,ab. or factorial*.ti,ab. or assign*.ti,ab. or allocate*.ti,ab. or volunteer*.ti,ab. or 'controlled study'.ti,ab. or 'major clinical study'.ti,ab. or 'clinical article'.ti,ab. or 'single arm trial'.mp. or singl*.ti,ab. or 'single-arm'.ti,ab. or 'single arm'.ti,ab.	10,185,012
11	exp longitudinal study/ or exp retrospective study/ or exp prospective study/ or exp cohort analysis/ or exp cross-sectional study/ or exp cohort analysis/ or exp observational study/ or (longitudinal study or retrospective study or prospective study or cohort$ or follow up or cross-sectional study or cross sectional study or followup study or observational study or registry or registries or real world or cross sectional or claims database or electronic health record$ or EHR or electronic medical record$ or EMR$ or RWE).ti,ab.	10,235,117
12	9 and (10 or 11)	5830
13	(case report or case series or woman or man or child or adolescent or female or male or boy or girl or infant).ti.	2,054,193
14	case reports/ or case study/ or case report$.jx. or case report$.jw.	2780848
15	(Ephemera or "Introductory Journal Article" or News or "Newspaper Article" or Editorial or Comment or Overall).pt. or in vitro Techniques/ or in vitro study/ or (commentary or editorial or comment or mice or rat or mouse or animal or murine).ti.	7,033,403
16	review.pt. not (systematic or (meta and analy*) or ((indirect or mixed) and 'treatment comparison')).ti,ab.	5,913,781
17	or/13-16	16,724,357
18	12 not 17	5037
19	18 not conference abstract.pt.	1985
20	(American Association for Cancer Research or AACR).nc,cg,cf.	112,768

21	(American Society of Clinical Oncology or ASCO).nc,cg,cf.	72,229
22	(American Society of Hematology or ASH).nc,cg,cf.	89,430
23	(European Hematology Association or EHA).nc,cg,cf.	27,518
24	(European Society for Medical Oncology or ESMO).nc,cg,cf.	37,025
25	or/20-24	338,970
26	limit 25 to yr="2016 -Current"	194,638
27	18 and 26	2019
28	19 or 27	4004
29	limit 28 to english language	3941
30	(202304$ or 202305$ or 202306$ or 202307$ or 202308$ or 202309$ or 202310$ or 202311$ or 202312$).dc. or (202313$ or 202314$ or 202315$ or 202316$ or 202317$ or 202318$ or 202319$ or 20232$ or 20233$ or 20234$ or 20235$).em.	1,861,288
31	(202304$ or 202305$ or 202306$ or 202307$ or 202308$ or 202309$ or 202310$ or 202311$ or 202312$).ed,dt.	1,234,980
32	29 and 30	461
33	29 and 31	102
34	32 or 33	563
**35**	**remove duplicates from 34**	**470**

Supplementary Table 2. PICOS screening criteria.*

**Population**	Adults ≥18 years of age with RRMM
**Intervention**	Anti-CD38-based therapy retreatment including: Daratumumab; isatuximab
**Comparison**	Any or none
**Outcomes**	Efficacy/effectiveness outcomes of retreatment with anti-CD38-based retreatment:<br>• OS; PFS/PFS2 (investigator or independent review committee); ORR; CR; durability of CR; stringent CR; PR; VGPR; MR; SD; PD; TTP; time to treatment failure; DOR; MRD negativity<br>In studies that report these outcomes, the following are also of interest where reported:<br>• Efficacy/effectiveness of the prior anti-CD38-based therapy (as assessed using any of the listed measures for assessing retreatment)<br>• Reasons for discontinuation of prior anti-CD38-based therapy<br>• Time from prior anti-CD38-based therapy
**Study design**	Interventional trials (randomized controlled; single-arm, non-randomized); observational studies

*To be eligible, studies with data unstratified by previous anti-CD38 exposure had to report results for a minimum of 80% of patients with such exposure.

CR, complete response; DOR, duration of response; MR, minimal response; MRD, minimal residual disease; ORR, overall /objective response rate; OS, overall survival; PD, progressive disease; PFS, progression-free survival; PICOS, Population, Intervention, Comparator, Outcome, Study Design; PR, partial response; RCT, randomized controlled trial; RRMM, relapsed/refractory multiple myeloma; SD, stable disease; TTP, time to progression; VGPR, very good partial response.

Supplementary Table 3. Patient characteristics reported across studies.

**Characteristic**	**Total number of studies**	**Total number of patients**	**Range across studies**
Age*	18	1896	Median 61–74 years
Male	15	1562	35–77%
Race	4	102	
White			53–86%
Black or African American			6–37%
Disease type	11	965	
IgG			40.6–72.2%
IgA			0–26.2%
Disease stage	14	1209	
Stage I			5–42%
Stage II			16–61%
Stage III			6–56%
High-risk cytogenetics[†], %	17	1650	11–81%
Number of prior lines of therapy	17	1763	Median 3–7 lines
Follow-up duration	12	1624	Median 2–53 months
Refractoriness	21	2367	
Double-refractory			3–100%
Triple-refractory			11–92%
Penta-refractory			7–67%

*Only studies reporting medians are included here. [†]The definition of high risk varied between studies. Ig, immunoglobulin.

Supplementary Figure 1. PRISMA diagram.

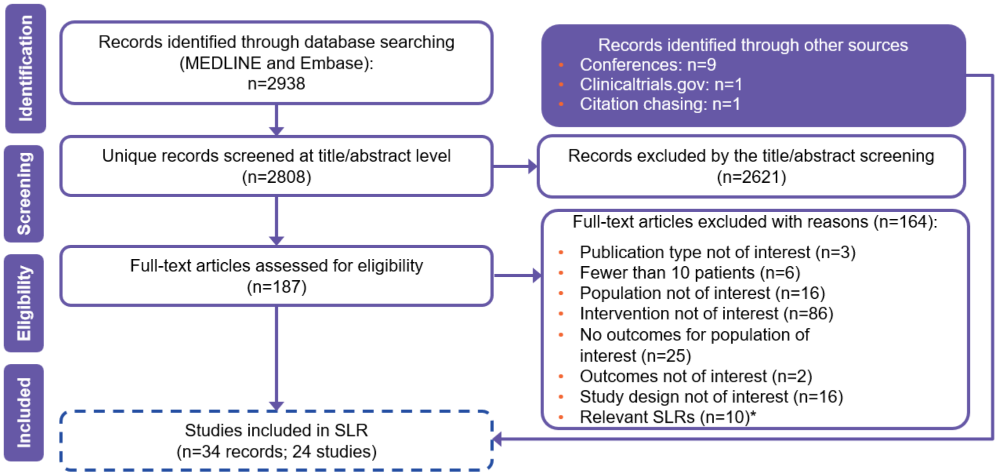

*Relevant SLRs were used for citation identification but were not included in this review to avoid double-counting data cited in both primary study records and the published SLRs.

PRISMA, Preferred Reporting Items for Systematic reviews and Meta-Analyses; SLR, systematic literature review.

Supplementary Figure 2. Median PFS in patients receiving anti-CD38-based retreatment and patients who were anti-CD38-based treatment naïve.

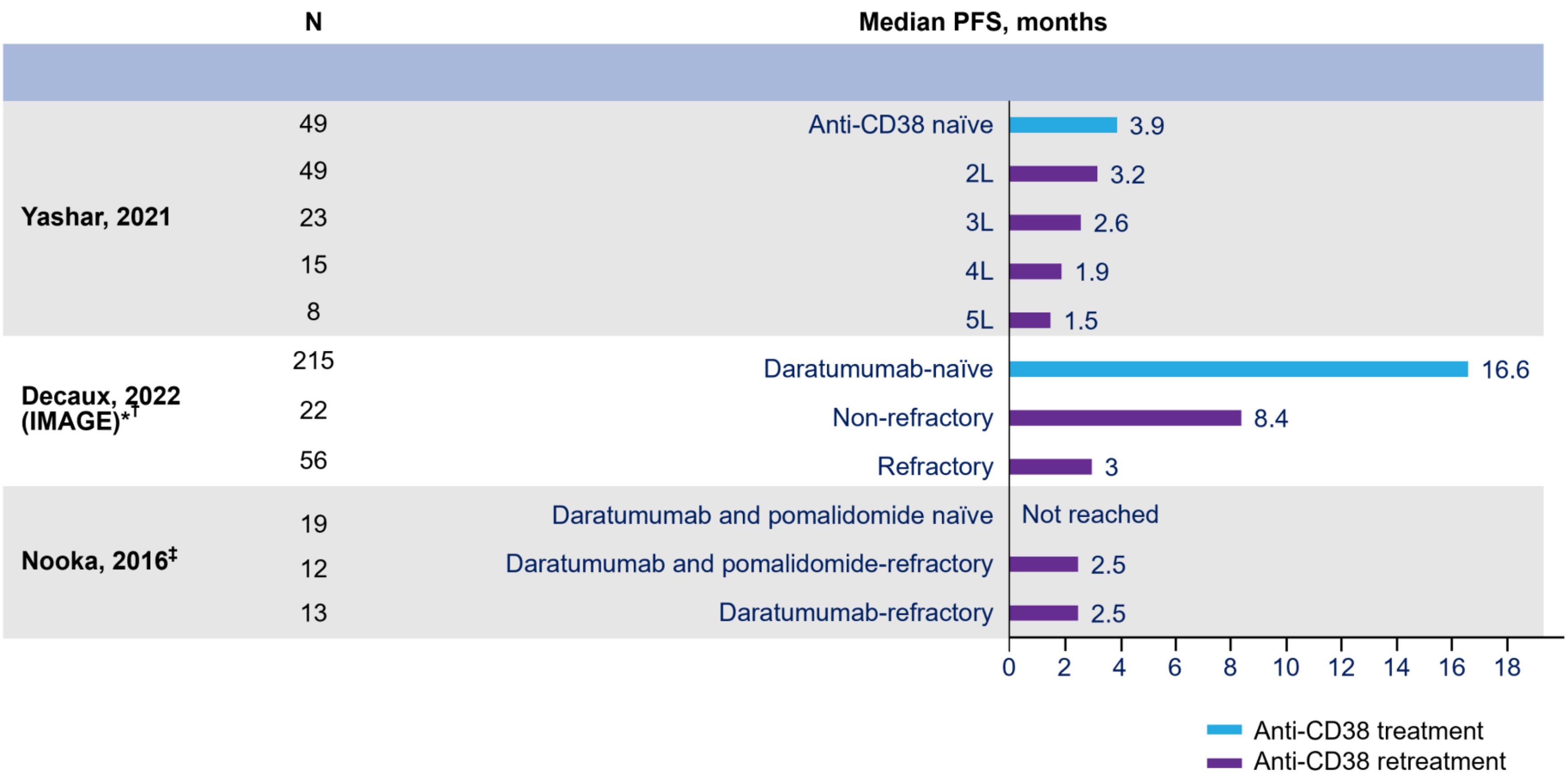

*Isatuximab plus pomalidomide plus dexamethasone treatment/retreatment.
†Proportion of anti-CD38 refractory patients was only reported for Decaux 2022; 28% of patients were daratumumab-refractory.
‡Daratumumab plus pomalidomide treatment/retreatment.

2/3/4/5L, second/third/fourth/fifth line of therapy; Carf, carfilzomib; Dar, daratumumab; Dex, dexamethasone; Isa, isatuximab; NR, not reported; PFS, progression-free survival; Pom, pomalidomide.

Supplementary Figure 3. ORR by (A) type of response in RWE studies and (B) in patients receiving anti-CD38-based retreatment and patients receiving anti-CD38-based treatment for the first time.

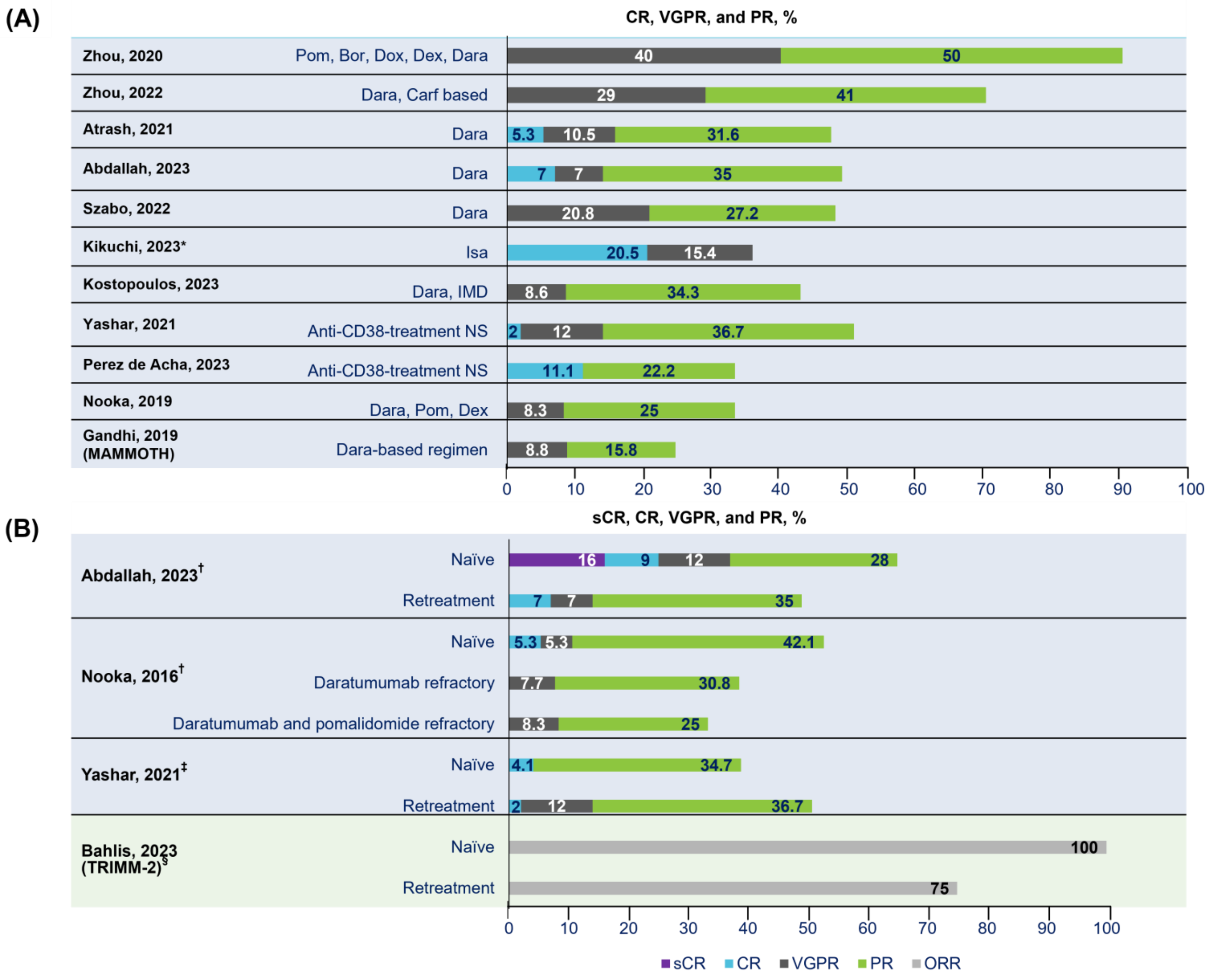

*Percent of patients with partial response was not reported in the publication.
† Daratumumab-based treatment/retreatment.
‡ Anti-CD38-based treatment/retreatment.
§ Daratumumab plus talquetamab treatment/retreatment.

Bor, Bortezomib; Carf, carfilzomib; CR, complete response; Dara, daratumumab; Dex, dexamethasone; Dox, doxorubicin; Isa, isatuximab; NS, not specified; ORR, overall response rate; Pom, pomalidomide; PR, partial response; Talq, talquetamab; RWE, real-world evidence; sCR, stringent complete response; VGPR, very good partial response.
